# Current and Future Perspectives of Liaison Psychiatry Services: Relevance for Older People’s Care

**DOI:** 10.3390/geriatrics1010007

**Published:** 2016-03-03

**Authors:** Elizabeta B. Mukaetova-Ladinska

**Affiliations:** Institute of Neuroscience, Newcastle University, Newcastle upon Tyne NE4 5PL, UK; Elizabeta.Mukaetova-Ladinska@ncl.ac.uk

**Keywords:** liaison psychiatry, older adults, delirium, dementia, liaison service models

## Abstract

A large number of people admitted to medical wards have co-morbid mental health problems, and these predominantly include depression, dementia and delirium. An additional one third of medically ill patients remain in hospitals with undetected and, therefore, undiagnosed mental health problems. The comorbidity of mental and physical illnesses leads to poor health outcomes, prolonged inpatient stays and use of inpatient resources, involvement of various affiliated health services, introduction of medications and discharge to long-term facilities, including residential and nursing 24-h care, increased both readmission rates and mortality. The establishment of Liaison psychiatry services to meet the needs for people with mental health problems admitted to medical wards is a priority for many acute health Trusts. This has an economical background in terms of cost-savings, especially in relation to the older adults, with decreasing readmission rates and quicker hospital discharges. In the current review, we address the latest policies regarding Liaison psychiatry services; especially those for older people with dementia and delirium, and discuss their future shaping.

## 1. Introduction

A large number of people admitted to medical wards have co-morbid mental health problems. These predominantly include depression [1,2,3], organic mental health problems, such as dementia [4,5] and delirium [6,7]. An additional one third of medically ill patients remain in hospitals with undetected and, therefore, undiagnosed mental health problems [8]. It is estimated that a half of all inpatients suffer from a mental health condition [9], though this figure may well be higher [10]. The comorbidity of mental and physical illnesses leads to poor health outcomes in terms of prolong inpatient stays, longer use of inpatient resources, e.g., rehabilitation, involvement of various affiliated health services, medication, discharge in long-term facilities, including residential and nursing 24-h care [11,12], and increased mortality [13,14,15]. Not surprisingly, the latter findings result in increased health care costs [16] that are not only confined to the inpatient stay, but also to the increase in outpatient attendance [17,18]. 

## 2. Mental Health in Medically Ill: Review of Policies

Over the last 10 years, a number of publications have raised the importance of the treatment of mental health in medically ill people. Thus, the Royal College of Psychiatrist document Who Cares Wins [19] provides a comprehensive overview about the value of mental health care for older people in general medical setting. Similarly Department of Health documents Everybody’s Business [20] and No Health without Mental Health [21] also stress the importance of physical and mental health co-morbidity, via improving the mental health care for people with long-term physical conditions [21]. The latter document also provides an example of a “collaborative care” approach when treating depression in people with type 2 diabetes in primary care. In addition, it highlights this approach alone to have a potential to save the National Health Service (NHS) and Social Care Service up to 3.4 million in a four-year period, with a further £12 million of benefits to individuals from improved productivity. 

Since mental health problems co-exist with a number of chronic and acute health conditions (e.g., depression and heart problems, depression and cancer, delirium and acute infections, *etc.*) it is not surprising that mental health co-morbidities contribute to a substantial increase in physical health care, amounting up to £12.5 billion/year extra expenditure on physical health services [17,18]. This is largely due to the growing ageing population with chronic illnesses (e.g., osteoporosis, cancer and dementia) who requires long-term health care involvement, continuous need of introducing new technologies and higher patient expectations [22]. For a typical 500-bed general hospital, this translates to extra costs of £25 million/year [9]. It is estimated that only by reducing the inpatient stay will save NHS at least £5 million/year/hospital [9]. The search for additional cost-savings to the National Health Service has identified that reducing the re-admission rates [23] and quicker discharge [24] for older adults with various physical and mental health problems (e.g., dementia) will contribute to further cost savings of £6.5 million in local areas, amounting up to £300 million/year across England [24]. These figures argue that cost-savings are achievable, especially if the targeted services are those that provide healthcare for older adults with chronic mental health problems. A recent work by Department of Health [22] identified the need for the NHS to save £15–£20 billion by the end of 2013/14, so that the savings can be reinvested in the health service to deliver quality improvements. This resulted in setting up a national programme of work streams to support clinical teams and NHS organizations that focus on three aspects, long-term conditions, urgent care and end-of-life care. 

## 3. Provision for Liaison Psychiatry Teams Working in General Medical Setting

Mental health diseases alone or co-existing with physical illnesses cross cut across all the above three work: mental health problems are chronic, some of them require urgent attention and also need end-of life care (e.g., dementia and delirium). Liaison psychiatry, thus, needs to play a major role in the latest Department of Health initiative. Liaison psychiatry has developed over the last two to three decades in response to the overlap between mental and physical problems, and the organizational separation between the mental and physical health services. Often established by enthusiastic mental health professionals, the provision of clinical services by the Liaison teams is rather variable across the United Kingdom [25,26].

Since Liaison psychiatry teams are involved not only in providing advice, treatment, follow-up and successful discharges of inpatients with polycomorbidities, but also with education of the medical staff how to look after their medically ill with co-existing mental health problems (Table 1), it is of no surprise that the provision of Liaison psychiatry in the United Kingdom has been labelled as “unacceptable”, resulting in a consensus that Liaison psychiatry provision must be addressed in order for the NHS to be more effective and efficient [25]. According to the Academy of Medical Royal Colleges, acute psychiatric Liaison services are an essential part of a whole acute service drawing in the specialist expertise and facilitating collaboration “within, between, and beyond hospitals” [25]. Furthermore, the Liaison psychiatry service activity can contribute to new ideas and even solutions on how to improve efficiency and quality of care for subjects with polycomorbidities [22]. 

## 4. The Future of Liaison Psychiatry Services

The investment in development of new and expansion of currently available Liaison psychiatry services is seen as a must in the NHS development [27]. In future years, the accent of Quality, Innovation, Productivity and Prevention activity will be placed on delivering additional efficiency savings and quality improvements that will require the NHS to “focus on delivering transformational change through clinical service redesign” [27], including Mental Health. The latter has also been reiterated in the latest Commissioning and Quality framework goals, naming as priorities the improvement of dementia and delirium care, and improving diagnosis in mental health [28]. The document also clearly states the role of Liaison services in establishing dementia diagnosis and appropriate follow-up for older people with dementia identified on acute medical wards. 

**Enhanced and comprehensive Liaison services.** All the above policy papers clearly identify the specialist role of the Liaison services for older adults in medical setting. However, we still do not have a clear guidance of what Liaison service model should be in order to meet the medical settings’ real needs. In a recent editorial, Sharpe [29] argued that one model (an ageless Liaison service) should fit all. This is in sharp contrast to Aitkin *et al.* document on the Liaison service models [26], that identified four distinct Liaison service provisions (Table 2), all including older people’s mental health needs, with the “Enhanced 24 h” service having more medical input for the older person. The latter document also provided an informative overview of the current Liaison services in the UK, with only 8% implementing comprehensive (2%) or enhanced (6%) Liaison services, whereas around 60% of the service were described as inadequate in relation to quality of care or outcomes [26]. 

The enhanced Liaison services correspond to the Birmingham Rapid, Assessment, Interface and Discharge (RAID) specification highlighted as an example of good practice. The Birmingham Rapid Assessment, Interface and Discharge service has attracted lots of attention in the light of its favorable economic evaluation with the biggest economic benefits coming from interventions in older adults [23]. This model is considered as a golden standard by some NHS organisations for development of similar services. However, in doing so, many of them overlooked the fact that the success of the Rapid, Assessment, Interface and Discharge model was linked to the parallel development of new model of intermediate care for the city of Birmingham, spanning both intermediate care inpatient facilities (for patients requiring short-stay interim, rehabilitation or respite care and assessment), and intermediate care services (consisting of 43 integrated multidisciplinary teams, each incorporating district nurses, case management, domiciliary physiotherapy, occupational therapy and community rehabilitation already in place for some parts of the city). Having these facilities undoubtedly contributed to quicker inpatients discharges, reducing inpatient (re)admissions, and providing more intensive community outreach for vulnerable subjects needing complex involvement of various services. 

In contrast to Birmingham, such facilities are rather limited for many of the citizens throughout the United Kingdom, thus arguing that the success of the Rapid, Assessment, Interface and Discharge-like Liaison models will be dependent on the availability of social support within the localities. Although currently many of the Liaison services call themselves Rapid, Assessment, Interface and Discharge, most of them fail the enhanced criteria, and in fact represent core 24 (14%) or core (39%) Liaison services instead [26]. Interestingly, within one year only, the second national Liaison psychiatry service survey in England showed a significant drop in both 24 h core and core services, to 5.6% and 12%, respectively, with subcore Liaison services (75%) replacing the more advanced Liaison psychiatry services in England [30]. Nevertheless, the overall impression is that the Liaison service development has progressed over the years, though they still remain understaffed, needing at around 500 additional consultants post to be created in order to provide a reasonable mixture of clinical services [30]. 

**Improving the core Liaison services**. Many of the newly established Liaison services are commissioned de novo, and are in a much better position to be shaped according to the established service specifications (i.e., working hours, staffing, clinical provisions, including specialist outpatient clinics, teaching and even inpatient clinical care). However, many well-established services seem not to have benefited from the latest investments in expanding and modernising of the Liaison services. The enhanced and comprehensive models of Liaison service provision all incorporate core models [26], and this should be a starting point in updating and improving the already established Liaison psychiatry services, pending on their local needs. Their up-date to enhanced and/or comprehensive models should be based around expansion on the older people services, including multidisciplinary and specialised outpatient clinics for delirium, dementia and behavioural problems, long hour’s provision for Accident and Emergency and acts of deliberate self-harm (Table 3). However, this service development may face an objective ‘trap’—many of the Liaison service users may be satisfied with the arrangements as they are, and would not like to invest further in their upgrade. This, in turn, can lead to additional pressure on the already saturated Liaison service provision that in the long run may result in abandoning some of the clinical services, maintaining only the essential, core, liaison arrangements.

**Out-of-hospital Liaison services provision:** Out-of-hospital provision represents an important extension of Liaison psychiatry, with the long-term development of Liaison psychiatry likely to lie ”primarily in the expanded provision of community-facing services” [23]. Providing Liaison psychiatry services at outpatient and primary care level has potential to reduce the number of referrals to secondary/tertiary care services, especially older people who suffer from long-term conditions. According to Parsonage et al [23], developing community-based collaborative care services with an integrated Liaison psychiatry component should be “a high priority for all clinical commissioning groups working with local providers” [23].

The “virtual ward” program (also referred to as Joint Emergency Teams) represents one of these community-based collaborative care services, where the input from Liaison psychiatry services can make a difference for the older and frail adults. The aim of the virtual wards is to provide multidisciplinary case management services to people who have been identified to be at high risks for emergency hospitalization, using a predictive, computerized, model. In addition, virtual wards also can be used to promote and support an early discharge for medically fit patients who wish to return home rather than remain in hospital, or assess adults in Accident and Emergency departments. Initially launched in 2006, virtual wards have now been introduced in several parts of the United Kingdom.

Virtual wards use the systems and staffing of a hospital ward, but without the physical building. They have close working relationships with social services, intermediate care facilities, and organizations, such as hospices, drug and alcohol service, hospital specialists and voluntary sector agencies. The identified at risk people are offered extra support, using the systems of a hospital ward providing them with a multidisciplinary case management, and thus avoid emergency hospital admissions. This approach is particularly useful for older adults with dementia, who face deterioration in their mental and physical health, including behavioral problems and higher death rate when admitted to hospitals [31]. 

The overall economic analysis of the pilot data to date is somewhat disappointing, with no evidence for an overall reduction in emergency hospital admissions, or mortality, though there was a reduction in elective hospital admissions and outpatient attendances [32]. One explanation for this is that the patients who receive this intervention have very complex illnesses, and thus represent a challenge for the NHS, in terms of both cost and quality of care provided. We cannot exclude the possibility that in the long-run this model of medical care may be cost effective, at least for some groups of users, e.g., older and frail individuals. In support of this is the evidence by the Greenwich multidisciplinary Joint Emergency Team, that reported avoiding 2000 unnecessary admissions, no delays in older people’s discharges, and savings of one million pounds from social care budget over 2.5 years’ time [33]. However, over the years, virtual wards philosophy may easily decent into standard (one-to-one) care management, and thus safeguarding the multidisciplinary nature of the intervention, ensuring the active involvement of General Practitioners, and multidisciplinary professionals needs to be represented [32]. The latter should also include an input from the Liaison psychiatry services catering for the needs of the frail older people, who in most instances are the most frequent users of virtual wards, due to complexity of their medical problems, e.g., falls, recent delirium episode requiring inpatient treatment, awaiting long-term care, or monitoring of change of medication, *etc.*

**Teaching and training**: The Liaison psychiatry services need to expand on teaching and training of junior colleagues and students, as well as introducing an academic component to the Liaison field. Many Acute Trusts have developed or are in a process of developing teaching materials for mental health for their staff. The input of Liaison psychiatry services is essential, in order to update the medical practitioners for the latest developments in the treatment of mental health issues, and adapt them for their clientele. A recent implementation of an innovative inter-professional teaching intervention (founded on medical education research findings in clinical setting) increased staff confidence in managing the confused older person on medical wards [34]), empowering staff to introduce relevant practice change. This argues that highly skilled medical educationalists with a background in Liaison psychiatry need to lead and develop educational programs to support and drive the change in dealing with mental health problems on medical wards. 

Except for a limited research work completed on service development and characterisation of the Liaison team for older adults [35], there is very little additional academic work in relation to the Liaison professional activity. Our pilot data on current scope of work by seven Liaison services in the UK indicates that education is one of the major gaps in the Liaison services [36] and needs to be urgently addressed in the light of the latest Commissioning and Quality framework for dementia and delirium. The latter requires working closely with the Acute Trusts to develop new programs to educate clinical and support staff about chronic and acute mental health problems older people may exhibit during their inpatients stays. The latter, similarly will require additional funding to deliver and share our knowledge. 

## 5. Conclusions

The question remains whether commissioning enhanced and/or comprehensive Liaison psychiatry services may help elevate the economic burden in the currently available medical care provision within the NHS. As Griffiths [37] states, the Liaison psychiatry services may not save the NHS on their own, but they certainly will help improve the quality of care and outcomes for numerous medically ill people, and thus fulfill at least some of the requirements as outlined in the Operating NHS Framework 2012/2013. However, the dynamics of the interplay between mental and medical health professionals may well be difficult, and require developing and nurturing effective teams, with understanding and stamina to deliver both clinical services and contribute towards the expected economical savings This is a process that has just started in many Mental and Acute Health NHS Trusts, and careful planning of these services is a prerequisite for the efficient delivery of care.

We still do not know what the key components and characteristics of a good Liaison service are. This is largely due to Liaison psychiatry services:

- being not well researched area (incomplete and inconclusive evidence based);
- being not easy for evaluative research (e.g. heterogeneous groups, complex interventions, lack of quality data and absence of metrics to measure relative performance) and
- having wide diversity of service models (assessment/management, various treatment).

The newly introduced Framework for Routine Outcome Measurements in Liaison psychiatry [38] should help at least with consistency of data collection and effective reporting of outcomes and their transparency in individual Liaison psychiatry services. This is a reasonable starting point to facilitate further discussions regarding quality of Liaison psychiatry services, and their further developments across the United Kingdom, with an aim to standardise the quality of clinical provisions. 

By 2020, 50% of the liaison services in the United Kingdom are expected to be Core 24 and their transformation is under way. However, this may prove to be a vulnerable solution to modernising the current NHS, in terms of the disparity between accessibility and quality of clinical service provision. Thus, there is a threat of lack of adequate senior clinical staff *versus* employment of lower grade nursing staff (working across specialties) and loss of specialty expertise, with transferring the clinical response to urgent and emergency clinical care, rather than responding to objective clinical pressure, *i.e.*, older people, who represent the largest inpatient’s group. Furthermore, the highly desirable incorporation of the Liaison psychiatry services within the acute medical milieu [39] may not necessarily result with the expected outcomes. Thus, in lieu of integration with medical services, the Liaison psychiatry services may not necessarily facilitate the efficient transition of clinical care from medical to mental health care environment. A more structured research perspective, incorporating a population based approach to determine the needs of service users and, thus, guide service development within different geographical areas [40] is needed. This, undoubtedly, will highlight the need for closer working partnerships with charities and care providers’ agencies, in order to foster effective multi-agencies collaborations and improve the health care workflow during hospital discharges and transition periods. 

There is very little information about the organisational structure of the liaison psychiatry services worldwide. The now 10-years old European survey reports great variability of the Liaison psychiatry service provision, with 54% providing 24-h clinical service and functioning as emergency mental health services within general hospital setting [41]. However, there is very limited information about the clinical services they provide. Furthermore, the lack of organizational developments of service provision makes the latest United Kingdom developments difficult to compare with those in Europe or worldwide. 

In conclusion, although Liaison psychiatry services may provide only modest improvement in health outcomes, they have a potential to result in significant savings in heath service costs. Their development, therefore, should be a priority for the NHS Trusts and the health systems worldwide.

## Figures and Tables

**Table 1:** Liaison Psychiatry Services for older adults: Quality of care.

1	Early detection of dementia
2	Behavioural and psychological symptoms of dementia (BPSD)
3	Detection and management of delirium
4	Mood disorders (depression, bipolar disorder)
5	Anxiety
6	Insomnia
7	Suicidality
8	Anorexia
9	Advocates/social issues
10	Pharmacological and non-pharmacological treatments of psychiatric syndromes
11	24-h care
12	Ethical issues (e.g. competence and capacity decisions)
13	Medico-legal assessments
14	Education and training

**Table 2:** Liaison service models (after Aitkin *et al.*, 2014 [26]) Abbreviation: ME, myalgic encephalomyelitis.

Models	Characteristics
Core	Working or extended hours only; serves acute health care systems with or without minor injury or emergency department environments where there is variable demand across the week.
Core 24	24 h, seven days a week; hospital based in urban or suburban areas with a busy emergency department. This model mainly serves emergency and unplanned care pathways.
Enhanced 24	24 h, seven days a week, with extensions to fill local gaps in service and some outpatient services; additional expertise in addictions psychiatry and the psychiatry of intellectual disability. Demography and demand may suggest additional expertise with younger people, frail elderly people or offenders, crisis response or social care. This may extend to support for medical outpatients. This model mainly serves emergency and unplanned care pathways but extends to support elective and planned care pathways where mental health problems co-exist.
Comprehensive	24 h, seven days a week, enhanced with inpatient and outpatient services to specialties at major centres. Required at large secondary care centres with regional and supra-regional services. Additional specialist consultant liaison psychiatry, senior psychological therapists, specialist liaison mental health nursing, occupational and physiotherapists. They support inpatient and outpatient areas such as neurology, gastroenterology, bariatric surgery, plastic and reconstructive surgery, pain management and cancer services. They may support other condition specific elements such as chronic fatigue / ME and psychosexual medicine. They may include specialist liaison psychiatry inpatient beds. This model serves emergency and unplanned care pathways as well as elective and planned care pathways where mental health problems co-exist.

**Table 3:** Recommendations for development of Liaison psychiatry services.

New Services	Old (Established) Services
Start with a rapid response generic service, and then consider add-ons.	Consider add-ons (e.g., multidisciplinary outpatient clinics, substance misuse clinics, *etc.*). Long hours provision only for Accident and Emergency and Deliberate Self-Harm activity.
Focus on complex and costly cases.	Further develop outpatient liaison and multidisciplinary clinics, e.g., delirium clinics, dementia/behavioural problems follow-up, links with community services, *etc.*).
Core work in medical wards and Accident and Emergency.	Extend work to Accident and Emergency for all ages
An all ages service	Integrated Liaison service, to include children, adults and older adults
Work with older inpatients should be a top priority.	Expand on Liaison teams for older adults to expand on core clinical work, teaching and multidisciplinary and outpatient liaison clinics.
Emphasize education, training and supervision of general district staff - Spend half of time on education and training.	Expand on Liaison teams to incorporate teaching and training as part of their core professional activity.
Change culture of local care health system to response to and support development of Liaison psychiatry services	Ongoing assistance from local care health system(s) to support Liaison services.
